# Multi-locus sequence data illuminate demographic drivers of Pleistocene speciation in semi-arid southern Australian birds (*Cinclosoma* spp.)

**DOI:** 10.1186/s12862-016-0798-6

**Published:** 2016-10-22

**Authors:** Gaynor Dolman, Leo Joseph

**Affiliations:** 1 Molecular Systematics Unit, Western Australian Museum, Locked Bag 49, Welshpool DC, WA 6986 Australia; 2 Australian National Wildlife Collection, CSIRO National Research Collections Australia, GPO Box 1700, Canberra, ACT 2601 Australia; 3 Australian Centre for Evolutionary Biology and Biodiversity, School of Biological Sciences, University of Adelaide, Adelaide, SA 5005 Australia

**Keywords:** Australian birds, Eyrean Barrier, Semi-arid, Phylogeography, Coalescence, Demographic history, Speciation, Southern Australian biogeography

## Abstract

**Background:**

During the Pleistocene, shifts of species distributions and their isolation in disjunct refugia led to varied outcomes in how taxa diversified. Some species diverged, others did not. Here, we begin to address another facet of the role of the Pleistocene in generating today’s diversity. We ask which processes contributed to divergence in semi-arid southern Australian birds. We isolated 11 autosomal nuclear loci and one mitochondrial locus from a total of 29 specimens of the sister species pair, Chestnut Quail-thrush *Cinclosoma castanotum* and Copperback Quail-thrush *C. clarum*.

**Results:**

A population clustering analysis confirmed the location of the current species boundary as a well-known biogeographical barrier in southern Australia, the Eyrean Barrier. Coalescent-based analyses placed the time of species divergence to the Middle Pleistocene. Gene flow between the species since divergence has been low. The analyses suggest the effective population size of the ancestor was 54 to 178 times smaller than populations since divergence. This contrasts with recent multi-locus studies in some other Australian birds (butcherbirds, ducks) where a lack of phenotypic divergence was accompanied by larger historical population sizes. Post-divergence population size histories of *C. clarum* and *C. castanotum* were inferred using the extended Bayesian skyline model. The population size of *C. clarum* increased substantially during the late Pleistocene and continued to increase through the Last Glacial Maximum and Holocene. The timing of this expansion across its vast range is broadly concordant with that documented in several other Australian birds. In contrast, effective population size of *C. castanotum* was much more constrained and may reflect its smaller range and more restricted habitat east of the Eyrean Barrier compared with that available to *C. clarum* to the west.

**Conclusions:**

Our results contribute to awareness of increased population sizes, following significant contractions, as having been important in shaping diversity in Australian arid and semi-arid zones. Further, we improve knowledge of the role of Pleistocene climatic shifts in areas of the planet that were not glaciated at that time but which still experienced that period’s cyclical climatic fluctuations.

**Electronic supplementary material:**

The online version of this article (doi:10.1186/s12862-016-0798-6) contains supplementary material, which is available to authorized users.

## Background

Understanding the effect that cycles of cooling and warming during the Pleistocene had on the diversification and distribution of fauna and flora is a central theme in evolutionary biology [1]. The impact of the Pleistocene is particularly well understood in the temperate northern hemisphere [1]. It is widely accepted that species underwent range contractions, persisting in one or few refugia during glacial maxima. During warmer interglacials, ranges expanded opening up potentially novel habitats and providing opportunity for secondary contact and gene flow [1]. The effect that these isolation events and shifts in distribution had on the diversification of species varies across taxa, some species having diverged and others remained cohesive [2]. A largely unanswered central question is which processes drove these varying outcomes [2]? When speciation did occur, was it accompanied by increased genetic drift due to large reductions in population size when in contracted refugia, colonisation of newly suitable habitat, or divergent selection? If these processes acted in concert, how might their relative contributions be teased apart?

These questions are especially challenging in understanding the effect of Pleistocene cycles of cooling and warming on diversification in less studied areas such as unglaciated regions of the world, including arid or semi-arid regions (e.g., [3, 4]). Recently, in the context of southern and central Australian temperate, dry, semi-arid and arid woodland habitats, Kearns et al. [5] demonstrated that arid-adapted populations of Grey Butcherbirds *Cracticus torquatus* underwent range expansions during Pleistocene glacial maxima due to the expansion of the arid zone at that time. They based these conclusions on a robust multi-locus molecular dataset together with environmental niche models for butcherbirds (*Cracticus* spp.). In other southern Australian semi-arid fauna, the large and vagile Western Grey Kangaroo (*Macropus fuliginosus*) lost genetic diversity as its range expanded from south western Australia to the east according to analysis of microsatellite data [6].

Two recent studies are particularly pertinent to this issue. One, a multi-species hierarchical approximate Bayesian computation (hABC) approach found that 26 of 32 Australian avian populations showed concordant signatures of population expansion just prior to the Last Glacial Maximum (LGM) (at 35,225 years ago; 95 % quantiles 18,963–67,545) [7]. The 26 populations of birds are ecologically and phylogenetically diverse and inhabit a variety of different biomes. So the response appears to be a general one, across mesic, monsoon tropical, semi-arid and arid environments but some questions remain. Did those 26 population expansions follow significant contractions or were they simply expansions from already large populations into newly available habitat (or a combination across the 26 taxa)? Was speciation or, at least, morphological differentiation a more likely outcome if populations contracted substantially? The timing of this co-expansion is concordant with the expansion of the western populations of the Grey Butcherbird study (25,000 to 30,000 years) [5]. Species distribution modelling in [5] showed that the population expanded from an already large distribution. Other species of butcherbird showed slow but steady increases from before this time (eastern Grey Butcherbird) or maintained stable populations (Silver-backed and Black-backed Butcherbirds (*C. argenteus*, *C. mentalis*, respectively)).

A second, earlier study applied hABC to mitochondrial DNA of an assemblage of ten southern Australian semi-arid zone birds, three of which were a subset of species studied in [7]. It focussed on how many divergence events were necessary to explain the data and revealed two to three separate divergence events having affected them during the mid-late Pleistocene; these involved the Nullarbor and/or Eyrean Barriers [8]. Summary statistics of population expansion for individual taxa further revealed varying patterns among these and two further species (or species pairs). The presence and absence of signatures of population expansion varied across species and parts of species ranges [9]. Notably, the analyses highlighted varying degrees of morphological differentiation among the species relative to each other, and suggested different evolutionary forces operating on phenotype in broadly co-distributed species.

Multi-species mitochondrial DNA (mtDNA) studies as discussed above benefit from increased statistical power gained from pooling data into a single analysis [10]. They can accommodate coalescent, mutational and demographic variance associated with individual species and, especially for single loci, high variance of estimated parameters [11]. However, mtDNA can be prone to departures from neutral equilibrium due to effects of selection or migration-drift disequilibrium (either undetected because of confounding processes or untested), which leads to errors in estimates of demographic parameters [12]. These effects may be amplified in species with complex social structures inhabiting heterogeneous environments, as in the White-browed Babbler *Pomatostomus superciliosus* [13]. Pavlova et al. [14] also showed that in the Eastern Yellow Robin *Eopsaltria australis* high intraspecific mtDNA divergences can be misleading and unrepresentative of the evolution of the genome and historical demography. It was argued that the mtDNA in that case was under strong environmental selection despite ongoing nuclear gene flow [14, 15]. These issues can only be uncovered by jointly analysing multiple loci.

Our focus here is to examine the historical demography of the speciational history of two sister species of southern Australian birds, the Chestnut Quail-thrush *Cinclosoma castanotum* and the Copperback Quail-thrush *C. clarum* (sensu [9]). *C. clarum* and *C. castanotum* are semi-arid adapted, largely terrestrial species which inhabit fairly dense bushy shrubs and undergrowth of mallee scrub, *Acacia* scrubs, dry sclerophyll woodland, heath, and native pine [16]. Toon et al.’s [17] exploration of phylogenetic relationships within *Cinclosoma* using multiple nuclear markers provides biogeographic context for this study. *Cinclosoma* species diversified out of mesic habitat into semi-arid and arid environments. *C. clarum* and *C. castanotum* diverged 2.16 million years ago (mya), while the more arid adapted *Cinclosoma* species began to diversify 6.08 mya. Semi-arid and arid adapted groups diverged from a common ancestor 13.80 mya.

*C. clarum* and *C. castanotum* exemplify a situation where there is both substantial genetic structure (4.38 % net divergence in mtDNA between them [9]) and moderate phenotypic divergence accompanied by differing patterns of sexual dimorphism [9, 18]. When this work commenced, *C. clarum* and *C. castanotum* were treated as subspecies of a single species (hypotheses varied regarding location of subspecies’ limits; see [9]). Dolman and Joseph [9] proposed that the level of mtDNA divergence across the Eyrean Barrier coupled with concordantly patterned nuclear DNA (nDNA) [17] and taxonomic divergence warranted recognition of two species. Here, sampling of more individuals and more loci has various advantages. It will: i) enable assessment of population structure from multiple loci without a priori assignment of individual specimens to populations; ii) a more precise test of the model of divergence that is applicable to *C. clarum* and *C. castanotum*; iii) test whether gene flow between structured populations has been restricted since divergence; and key to the main objective of this study, iv) examine whether the process of divergence was accompanied by reductions in population size and/or population expansions; and if so v) estimate the timing of population expansion in divergent populations. In using the methodologies herein we also robustly test the species rank of *C. clarum* and *C. castanotum* using multiple-step, species delimitation methodology [19–21]: discovery of genetic discontinuities using GENELAND [22]; and coalescent divergence modelling using IMa2 [23] to estimate the level of gene flow between species since divergence. We stress that the present study addresses historical not contemporary demography. Therefore, our use here of 12 multiple loci alleviates the lack of sampling in some gaps such as the small one between the ranges of the two species across the broad region of the Eyrean Barrier and the larger one across much of the range of the subspecies *C. clarum fordianum*. Sampling in those gaps is of course more relevant to study of contemporary gene flow, and we are addressing this separately in a study that will necessarily use historical DNA from museum specimens.

## Methods

### Sampling

**Fig. 1:**
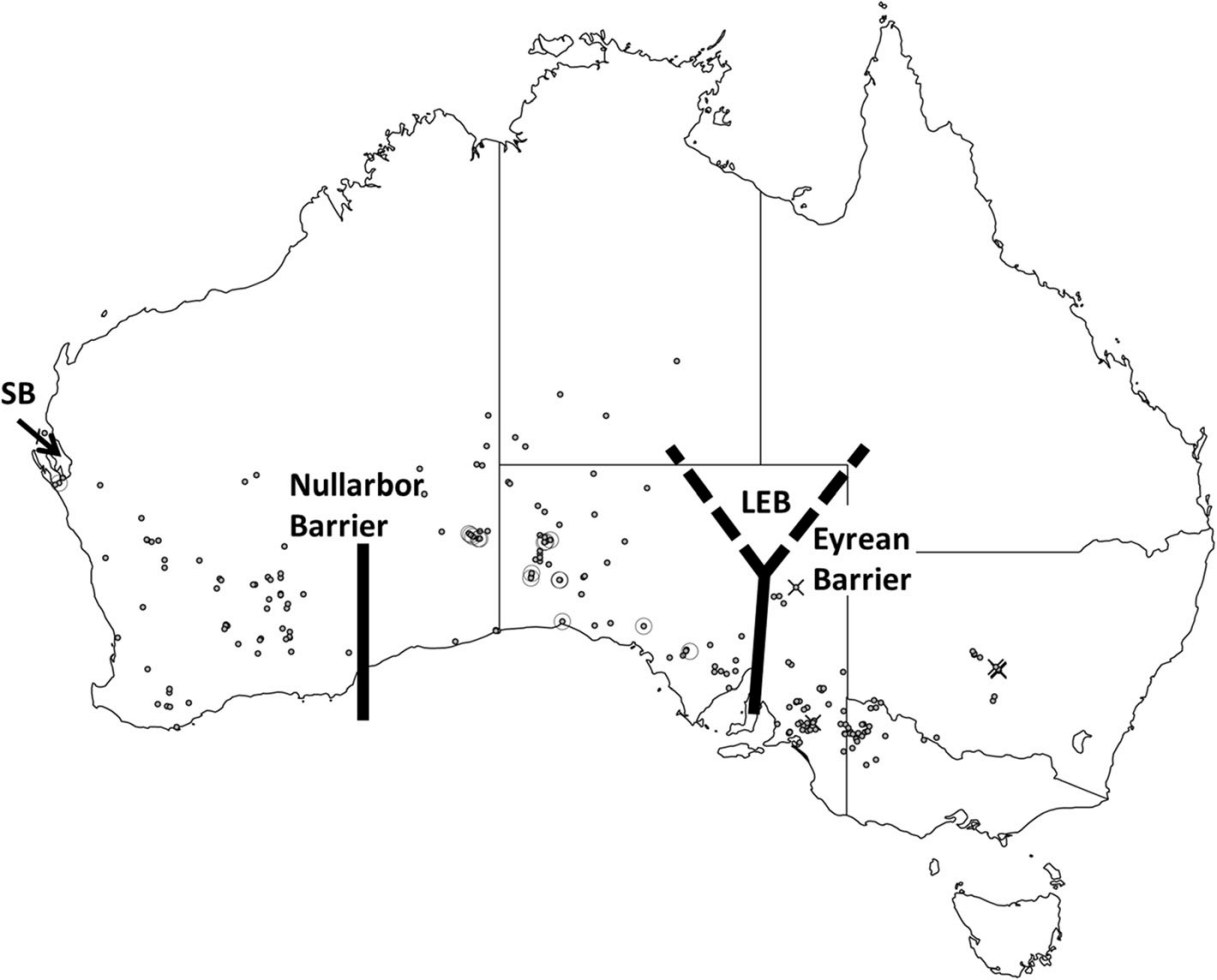
Map of Australia showing key geographic features, distribution information and sampling localities. Atlas of Living Australia data points for *C. clarum* and *C. castanotum* (*grey circles*) (filtered to represent museum specimens only) and specimens used in this study: *C. clarum* (*hollow circles*); *C. castanotum* (*crosses*). SB indicates Shark Bay and LEB indicates Lake Eyre Basin

A total of 29 *C. castanotum* (*n* = 7) and *C. clarum* (*n* = 22) liver tissues were sourced from the Australian National Wildlife Collection, CSIRO National Research Collections; Academy of Natural Sciences at Drexel University, Philadelphia; and South Australian Museum, Adelaide. Details are in Additional file 1: Table S1. Localities of tissue samples used in this study, together with a guide to the general distribution of C. castonotum and C. clarum (localities of all preserved msueum specimens) are presented in Fig. 1.

### DNA sequencing

Total cellular DNA was extracted according to the salting-out method [24]. DNA sequences from the *ND2* gene region of the mtDNA genome were reported in Dolman and Joseph (2015; GenBank numbers KM280401-KM280429). Eleven autosomal nuclear loci were amplified using the PCR primers detailed in Table 1. PCRs were performed in 25 μl reactions containing 1× reaction buffer (Eppendorf), 0.2 mM dNTP, 0.2 μM each primer, 1 Unit HotMaster Taq DNA Polymerase (Eppendorf) which specifically has no requirement for additional MgCl_2_. Cycling conditions included an initial denaturation of 94 °C for 2 min followed by a touch-down protocol of 34 total cycles of 94 °C denaturation for 30 s, 55 °C to 48 °C annealing for 30 s, extension at 65 °C for 45 s, and a final extension of 65 °C for 5 min. PCR products were purified with Ampure magnetic beads (Agencourt), and sequenced directly in both directions. Autosomal nuclear DNA sequences acquired for this study have been deposited in GenBank (accession numbers KX913359-KX913664). Phases of DNA sequences containing insertions/deletions (indels) were separated manually from the DNA sequence chromatograms as described in Dolman and Moritz [25]. For the remaining sequences with multiple heterozygous sites, phase was determined using the program Phase 2.1.1 [26, 27]. To avoid systematic bias in downstream coalescent analyses, we included all best allele pairs that were consistently resolved at a probability of 0.6 or higher in independent runs [28].

**Table 1:** PCR amplicon data

Locus Name	Primer sequence 5′ to 3′	PCR Primer reference	Product length (bp)	Recombination-free alignment (bp)	Number of taxa
Mame-AL06	F-AGAAGAATCCGTGTGCCAAC R-ATGTTCAGCACAACCACAGC	[71]	431–436	432	24
Mame-AL08	F-GCAAGGAAAGGGCATATCAG R-CCTCCACTGGACCTTCACTC	[71]	546–547	309	25
*α-Globin2*	F-CGTGGACCCDGKCAACTT R-CACAAGAACTTGTCCAGGG	[71]	187–207	207	28
*GAPDH*	F-TCCACCTTTGAYGCGGGTGCTGG R-CAAGTCCACAACACGGTTGCTGTATCC	[71]	345–350	216	28
*GTP*	F-ACGAGGCCTTTAACTGGCAGCA R-CTTGGCTGTCTTTCCGGAACC	[71]	732–741	236	28
12884	F- AGATGATGGAACAGAAGAG R- GCTATGAGTATGTTCTTTG	[72]	464–467	467	25
15506	F –GCAGTGCTTCTTTGTGAGCC R- CATTTAGAAGCCAGCGATAAC	[72]	997	832	22
20454	F- GTCCTGTGCCTTGTGTATGA R- CATCTCACAGTATTCCAGGC	[72]	327	327	16
26698	F- CAGAGGATGCGGAAGATGG R- TGATACAGAACAGATGACCC	[72]	565	499	19
16751	F- TGTTTGGAGGAGCACAAGAA R- AAGTAGAGGCCTGTGGTTTG	[72]	807–811	238	27
23989	F- AGCGTTGGAGCTTTCTTCAT R- TTCAACCCAAGATTCATTCC	[72]	299–301	301	28
*ND2* (L5216/H6313)	F- GGCCCATACCCCGRAAATG R- ACTCTTRTTTAAGGCTTTGAAGCC	[73]	1019	1019	29

PCR primers details, size of amplicon, size of phased, recombination free segment used in analyses and number of taxa for each locus

### Number of populations and population assignment

Allelic data from 11 autosomal nuclear loci were analysed together with spatial data for each individual using GENELAND [22, 29]. We used GENELAND to estimate the number of populations (*k*) and their spatial distribution. GENELAND was chosen over other clustering models because of the clear benefits of using a spatial model in cases of diverging subpopulations, potentially subject to admixture [30]. The number of populations, *k*, was estimated from multiple runs with 10 million iterations and 10,000 thinning and 10 % burn-in generations. Results were produced (with *k* fixed) from multiple runs of 50 million iterations, 50,000 thinning and 10 % burn-in generations. Runs were repeated 5 times to ensure convergence on a single result. We tested for hierarchical population structure, such that populations identified in the first round of analyses were put into another round of analyses to estimate the number of populations (*k*) and their distribution.

### Tests of assumptions

The isolation with migration model implemented in the program IMa2 assumes that there has been no recombination or gene conversion within the genealogical history of independent loci [31]. We used DNAsp 5.10.01 [32] to perform the four gamete test and to find gene regions excluding recombinant events [33].

The Hudson-Kreitman-Aguade (HKA) test [34] was used to test for neutrality across the 12 loci. HKA tests were performed on recombination-free segments of nuclear loci and the entire mitochondrial locus with 10,000 simulations using the computer program HKA (Jody Hey, Rutgers University). Deviations between observed and expected levels of divergence between populations identified using GENELAND, were summed across loci and the probability from Chi-square distribution (22 degrees of freedom) was calculated. The mtDNA locus (*ND2*) was also subjected to the McDonald Kreitman test [35] across divergent lineages in DnaSP v5.0 [36].

### Population divergence model

DNA sequence data from 11 autosomal nuclear loci and one mtDNA locus from populations identified using GENELAND were fitted to the Isolation with Migration model using IMa2 [23]. For two populations the parameters estimated, scaled by the neutral mutation rate (μ, geometric mean across loci of mutation rate per year per locus), were θA, θ1, θ2, effective population diversity for the ancestral population, and two daughter populations since divergence; *m*1 and *m*2, directional migration rates; and *t*, time since divergence. Isolation with migration was implemented with the Infinite Sites model for 11 nuclear loci (recombinant-free segments) and HKY for *ND2*. The mutation rate for *ND2* was given as 2.96 × 10^−5^ mutations per locus (1019 bp) based on an evolutionary rate of 0.029 per million years for the *ND2* gene [37]. Mutation rates were not provided for nuclear loci. We assumed an average generation time of 2 years based on published data [38–40] Several, relatively short preliminary metropolis coupled runs (10 million steps) were required to optimize the number of chains, heating parameters, and maximum parameter limits. Broad parameter limits were subsequently refined. Twenty chains with geometric heating (0.975 for the first heating parameter and 0.84 for the second heating parameter) were found to maintain a sufficient level of swapping between chains (average 74.5 % between chain 0 and chain 1 and 62.9 % between 18 and 19). Seven final M-mode runs were run with different random seeds for 45 million steps, and burn-in of 1,000,000. Swap rates for genealogy: (branch, topology and time since most recent ancestor, mutation rate scalar, HKY Model Kappa parameter (for mtDNA) and effective sample sizes were all adequate. Results from these seven runs confirmed convergence upon parameter distributions. Plots of parameter trends gave no indication of non-random trends. Genealogies were combined from these runs and 300,000 were used in an L-mode run to estimate joint posterior density and the corresponding joint posterior parameter estimates and to conduct model tests to assess which, if any, model appropriately fits the data. To address concerns that non-neutral evolution of mtDNA may affect demographic parameter estimates, analyses were repeated omitting mtDNA, using relative mutation rates for nuclear loci from posteriors of mutation rate scalars from initial runs with mtDNA. IMa2 is reportedly robust to population sub-structuring [41], but to verify the situation with *C. c. clarum* and *C. c. fordianum*, analyses were also repeated omitting the two specimens potentially representing *C. c. fordianum* according to mtDNA. The scaled mutation rates so recovered for nDNA loci were thought to be reasonable estimates based on published avian nuclear mutation rates of between 0.34 × 10^−9^ and 1.20 × 10^−9^ [42]. All but one nuclear mutation rate (minimum = 0.18 × 10^−9^) were within the published range (maximum = 0.97 × 10^−9^; average = 0.56 × 10^−9^).

### Timing of population expansion post-divergence

Bayesian skyline plots [43] can estimate the timing of the most recent population expansion. Using a single locus, moving backwards in time beyond the initial bottleneck, information on polymorphisms is lost, and any further population fluctuations are unlikely to be detectable [44, 45]. Using multiple loci to estimate Bayesian skyline plots has the potential to “see past” at least one population expansion event. This of course depends on the number of loci sampled, and the size of the most recent bottleneck. Population size histories of *C. clarum* and *C. castanotum* were inferred using the extended Bayesian skyline plot (EBSP) [45] on 12 and 11 loci respectively, in BEAST v1.7.5 [46]. Substitution models, clock models and trees for each partition (locus) were unlinked. Substitution models were chosen to maintain consistency with IMa2 analyses, i.e. simplest model for nuclear loci (HKY) and HKY for *ND2*, and a strict molecular clock was used. The evolutionary rate for *ND2* was fixed at 0.029 substitutions per million years [37]. The nuclear locus Mame-AL08 was fixed at 0.547 × 10^−9^ substitutions as this was the closest mutation rate to the average across all 11 loci of calculated from mutation rate scalars in IMa2 (average = 0.560 × 10^−9^). All other nuclear loci were estimated using normal distribution priors around starting estimates of rates calculated from estimates from mutation scalar in the IMa2 analyses. Two individuals representing putative *C. clarum fordianum* according to mtDNA were not included in the EBSP analysis of *C. clarum* as this population structure may result in false signals of population declines [47]. BEAST was run for 500 million generations, sampled every 50,000 generations with a 10 % burnin. Effective sample sizes of posterior parameter estimates were monitored to be at least above 100 and stationarity of parameter estimates were checked across multiple runs.

## Results

### DNA sequencing

The length of DNA sequence data for 11 nuclear loci for 29 individuals (average = 25 individuals per locus) ranged from 187 to 997 bp (average = 527 bp) (Table 1). Eight of 11 loci have indels which could be aligned unambiguously. The first position of each indel was recoded as a segregating character for use in IMa2; otherwise all gaps following the first position were coded as missing data.

### Number of populations and population assignment

Phase resolved bi-allelic data were used to estimate the number of populations and to assign individuals to those populations. The peak of the posterior distribution in GENELAND for the number of clusters was *k* = 2. With *k* then fixed to *k* = 2, several runs were found to all converge upon the same population assignment. Population assignment is concordant with mtDNA differentiation at the Eyrean Barrier and therefore concordant with current species delimitation of *C. clarum* and *C. castanotum* [9]. All individuals were assigned to their respective populations either side of the Eyrean Barrier with a probability of 1.0, except for specimen ANWC B33368 (Australian National Wildlife Collection, CSIRO) from Tamala Station, south of Shark Bay, which was assigned to *C. clarum* with a probability of 0.99. Posterior densities for *k* = 1 to 4 clusters and maps of posterior probability to belong to cluster 1 or 2 are presented in Additional file 2: Figure S1. Hierarchical analyses within these two populations revealed no further clustering of populations. The high level of mtDNA divergence of two specimens (assigned to *C. clarum*) from *C. clarum* (one from Tamala Station, Western Australia, and one from Shed Tank, South Australia), suggest that these specimens, at least according to mtDNA, could represent the subspecies *C. clarum fordianum*, or gene flow from it, or incomplete sorting of mtDNA within the two recognized subspecies of *C. clarum.* As *n* = 2 and geographic sampling is sparse, there may be insufficient power to detect a second genetic discontinuity between *C. clarum clarum* and *C. c. fordianum* using GENELAND. We included these two specimens as *C. clarum* in coalescent analyses of divergence, supported by available mtDNA evidence suggesting that the *C. clarum clarum* - *C. clarum fordianum* subspecies level divergence is indeed more recent than the *C. castanotum* - *C. clarum* species level divergence. However, analyses of divergence were also repeated in three independent runs with these two specimens removed and results changed very little as presented in Table 3.

### Tests of assumptions

Recombination-free alignments of nuclear loci range from 207 to 832 bp (average = 369 bp) (Table 1). These recombination-free alignments were used in all downstream analyses. The Hudson-Kreitman-Aguade (HKA) test [34] used to test for neutrality across the 12 loci showed no significant deviation from neutrality: sum of deviations = 10.892; d.f. = 22; Chi Square *P* = 0.976.

The McDonald-Kreitman test [35] on the coding *ND2* gene showed evidence for purifying selection in divergent populations of *C. castanotum* and *C. clarum* (synonymous mutations: 37 fixed and 32 polymorphic; non- synonymous mutations:1 fixed and 9 polymorphic; *P* = 0.015; neutrality index = 10.406). While neutral mutations are still subject to genetic drift, deleterious mutations are removed. This will not affect downstream analyses except for lowering the overall mutation rate of *ND2*, possibly underestimating the timing of demographic events.

### DNA diversity

Recombination-free DNA sequence data revealed low to moderate levels of variation at the 11 nuclear loci. The number of polymorphic sites ranges from 2 to 11 (average = 6) and number of haplotypes ranges from 3 to 13 (average = 7) (Table 2). Average nucleotide diversity across all 11 loci is 0.236 % for *C. clarum* and 0.182 % for *C. castanotum* (Table 2). The average number of distinct haplotypes across all 11 nuclear loci is 6 for *C. clarum* and 3 for *C. castanotum* and average haplotype diversity is 0.492 and 0.493, respectively for *C. clarum* and *C. castanotum* (Table 2).

**Table 2:** Genetic diversity and divergence statistics

	*C. clarum*				*C. castanotum*				Divergence
Locus Name	Number of alleles	Number of distinct haplotypes	Hd	Pi	Number of alleles	Number of distinct haplotypes	Hd	Pi	Da
Mame-AL06	36	7	0.478	0.00173	12	3	0.318	0.00077	0.00185
Mame-AL08	36	4	0.383	0.00133	14	3	0.703	0.00285	0.00512
*α-Globin2*	42	6	0.509	0.00182	14	3	0.538	0.00335	0.00011
*GAPD*	42	8	0.663	0.00804	14	2	0.363	0.00169	0.00348
*GTP*	42	11	0.697	0.00424	14	5	0.505	0.00279	0.00055
12884	36	2	0.322	0.0007	14	2	0.143	0.00031	0.00007
15506	38	9	0.585	0.00094	6	4	0.800	0.00195	0.00006
20454	18	2	0.294	0.0009	14	4	0.758	0.00339	0.00208
26698	30	5	0.361	0.00117	8	2	0.250	0.00050	0.00002
16751	14	5	0.605	0.00299	14	3	0.385	0.00174	0.00385
23989	14	7	0.516	0.00211	14	2	0.363	0.00121	0.00006
*ND2*	22	17	0.97	0.00566	7	7	1	0.00729	0.04316

Number of alleles, number of distinct haplotypes, haplotype diversity (Hd), and nucleotide diversity (Pi), for each locus for i) *C. clarum* and ii) *C. castanotum*; and net divergence (Da), between *C. clarum* (including two specimens with putative *C. clarum fordianum* according to mtDNA) and *C. castanotum* for each locus

### Population divergence model

**Fig. 2:**
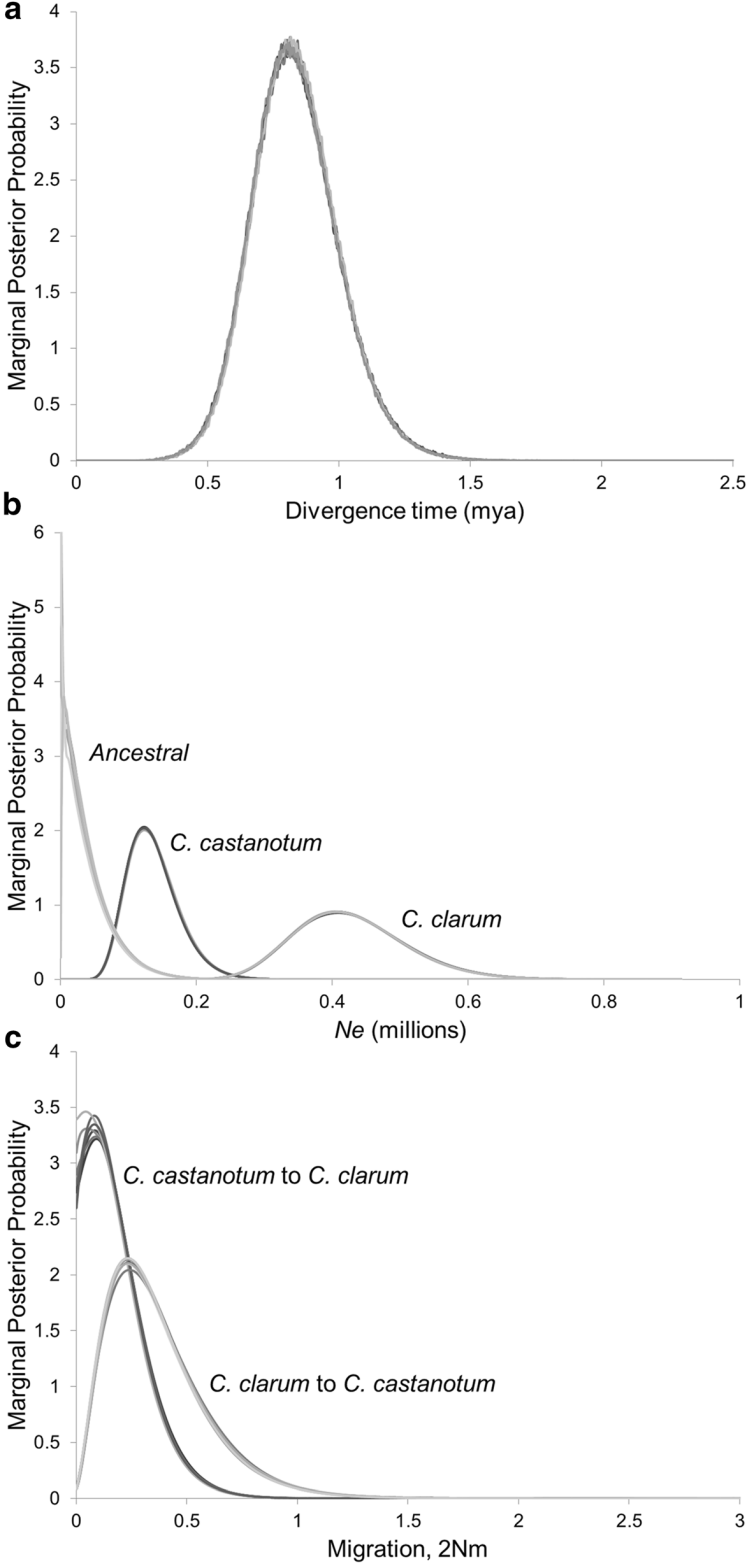
Multi-locus coalescent-based analyses of population divergence (IMa2). Marginal posterior density distributions from IMa2 analyses – seven independent MCMC analyses (M-mode) of DNA sequence data from one mitochondrial locus and 11 nuclear loci for the divergence of *C. clarum* and *C. castanotum* from their common ancestor. On a demographic scale, parameters are (**a**) time since divergence in years; **b** effective population size, *Ne*; and **c** effective number of migrants per generation, 2 Nm

Seven final M-mode runs of ca. 60 million steps each converged upon comparable peak parameter estimates (Fig. 2). Table 3 shows highest posterior parameter estimates and the corresponding 95 % upper and lower posterior density bounds for each parameter. Marginal posterior density distributions for each parameter are presented on a demographic scale in Table 4 and Fig. 2. The tree files for these seven runs were combined for an L-mode run. In the L-mode run based on 300,000 genealogies, the divergence model that attained the highest likelihood was the full model which allows migration estimates greater than zero and all population size parameter estimates to vary. However, models based on i) a coalescent migration rate of zero from *C. clarum* to *C. castanotum* or ii) equal migration rates could not be rejected (LLR =0.0 and 1.805, respectively; Table 3). Joint posterior estimates from the L-mode run for each of these three models are provided in Table 3. Effective population size (*Ne*) of the ancestral population is substantially reduced (2287 according to joint posterior estimates for the full model) compared with the two daughter populations (*C. castanotum* = 123,031 and *C. clarum* = 406,598). The timing of divergence is during the Middle Pleistocene according to the highest posterior estimate at 0.814 mya and 95 % lower and upper posterior density bounds, 0.514–1.180 mya. Migration since divergence (2 Nm) from west to east, i.e., *C. clarum* to *C. castanotum*, is not significantly different from zero. In contrast, migration since divergence from east to west (*C. castanotum* to *C. clarum*) is significantly greater than zero, but is nonetheless low (2 Nm <1.0; highest posterior estimate = 0.238; and 95 % lower and upper posterior density bounds = 0.020, 0.820). Repeating analyses without mtDNA produced essentially the same results. Note that the mutation rates for nuclear loci in these analyses were based on relative mutation rates compared with mtDNA in analyses including the full dataset. The importance lies in that the relative demographic parameter estimates are robust to omitting the mtDNA. Repeating analyses without the two specimens more suggestive genetically of being *C. c. fordianum* or affected by gene flow from it produced very low deviation from all peak parameter estimates.

**Table 3:** Parameter estimates from IMa2 population divergence model (scaled by mutation rate)

			Time	Population Size			Migration	
Scaled by mutation rate, μ	IMa2 Mode	LLR	tμ	4Nμ (*C. clarum*)	4Nμ (*C. castanotum*)	4Nμ (Ancestral)	mμ from *C. clarum* to *C. castanotum*	mμ from *C. castanotum* to *C. clarum*
Full dataset Highest Posterior	MCMC (M-mode)		0.551	2.226	0.675	0.013	0.091	0.200
HPD Lower			0.353	1.496	0.373	0.000	0.000	0.018
HPD Upper			0.803	2.249	1.169	0.593	1.438	0.725
1. Full model	Nested models (L-mode)	-	0.5437	1.869	0.779	0.001	0.000	0.952
3. Coalescent migration rate zero from *C. clarum* to *C. castanotum*		0.000		1.869	0.779	0.001	[0.00000]	0.952
2. equal migration rates		1.805		2.653	0.742	0.000	0.264	[0.264]
Putative *C. clarum ‘fordianum’* removed from analyses) Highest Posterior	MCMC (M-mode)		0.527	2.138	0.656	0.009	0.138	0.213
HPD Lower			0.333	1.406	0.358	0.000	0.000	0.016
HPD Upper			0.774	3.192	1.141	0.574	1.569	0.778

Presented here are highest posterior estimates and 95 % upper and lower posterior density bounds of parameters scaled by mutation rate from MCMC analyses (M-Mode); and joint posteriors and log likelihood ratios of nested models that could not be rejected, ranked highest to lowest (L-mode). M-mode analyses are averages of seven independent runs for the full dataset and three independent runs with putative *C. clarum fordianum* removed. L-mode analyses are based on a total of 300,000 genealogies combined from seven independent analyses

**Table 4:** Parameter estimates from IMa2 population divergence model (on a demographic scale)

		Time	Population Size			Migration	
Demographic scale	IMa2 Mode	*t*	*Ne* (*C. clarum*)	*Ne* (*C. castanotum*)	*Ne* (Ancestral)	2 Nm from *C. clarum* to *C. castanotum*	2 Nm from *C. castanotum* to *C. clarum*
Highest Posterior	MCMC (M-mode)	806,297	407,395	123,427	3332	0.074	0.235
HPD Lower		516,187	273,710	68,150	0	0.000	0.021
HPD Upper		1,174,815	594,399	213,858	108,530	0.454	0.815
Highest Posterior	Full-model (L-mode)	814,112	406,598	123,031	2287	0.081	0.238
HPD Lower		514,079	273,048	67,233	0	0.000	0.020
HPD Upper		1,180,004	593,204	213,590	109,310	0.454	0.820
MtDNA locus removed from analyses) Highest Posterior	MCMC (M-mode)	838,511	749,796	132,888	9491	0.108	0.304
HPD Lower		398,987	431,202	59,846	916	0.000	0.000
HPD Upper		1,344,210	1,401,586	264,858	166,032	0.522	1.356

Presented here are highest posterior estimates and 95 % upper and lower posterior density bounds of parameters scaled by mutation rate from MCMC analyses (M-Mode) and joint posteriors based on the full model in L-mode. M-mode analyses are averages of seven independent runs for the full dataset and three independent runs with the mitochondrial DNA locus (*ND2*) removed. L-mode analyses are based on a total of 300,000 genealogies combined from seven independent analyses

### Timing of population expansion post-divergence

**Fig. 3:**
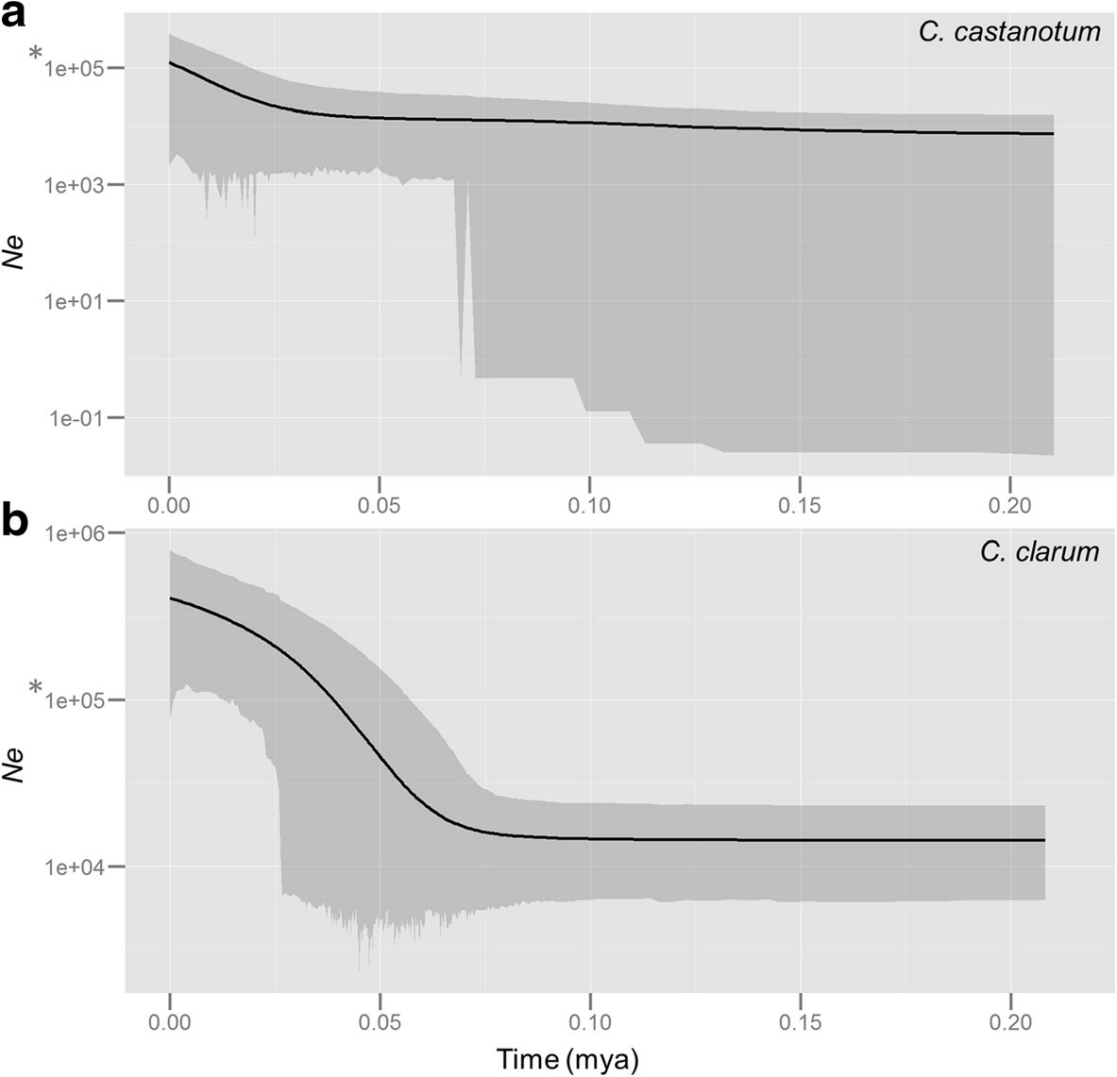
Extended Bayesian skyline plots (EBSP). Estimates of effective population size (Ne) over time (in millions of years) for (**a**) *C. castanotum* and (**b**) *C. clarum* based on combined nuclear and mitochondrial loci, using EBSP in BEAST v 1.7.5. The median posterior estimates are presented with the *shaded area* indicating the 95 % HPD. Effective population size, Ne is scaled to demographic scale as per IMa2 results and presented on a log10 scale. At time 0, Ne is comparable to current Ne in Table 3. *Indicates comparable Ne on EBSP plots with different scales

Signatures of population expansion are evident in both *C. clarum* and *C. castanotum. C. castanotum* began to increase in population size around 25 thousand years ago (kya) (HPD of ca. 35-0 kya), and *C. clarum* began to increase in population size around 75 ka (HPD of ca. 75-25 kya) (see Fig. 3).

## Discussion

We used multi-locus DNA sequence data to assess historical population structure within sister species of quail-thrush, *Cinclosoma clarum* and *C. castanotum*, from southern Australia. The genetic data defined spatial location of population clusters and individuals were assigned to these clusters. A critical result of our analyses, which we expand below, was that population structure was concordant with recently revised species limits [9] and congruent with the Eyrean Barrier being a major historical barrier in these species. The Lake Eyre Basin/Eyrean Barrier has long been hypothesized as a major phylogeographic break in birds [48–50] and indeed in quail-thrush, *Cinclosoma* spp. [18, 51]. This theory, based originally on morphology, has been supported by molecular data in other Australian birds, such as the Australian ringneck parrots (*Barnardius zonarius*) [52], the splendid fairy-wren (*Malurus splendens*) [53] the White-eared Honeyeater (*Nesoptilotis leucotis*) [8, 9] and White-browed Babbler [13].

Our data suggest that *C. clarum* and *C. castanotum* diverged across the Eyrean Barrier during the Middle Pleistocene. Inference from stratigraphic sequences within the distribution of *C. castanotum* suggests that their divergence likely coincided with a reduction of woodland and tall shrub cover during the Pleistocene [54]. This trend towards aridity in southern Australia was progressive and step-wise [55] and led to the presence of dry-land vegetation in south-eastern Australia at a site in western Victoria at 1.2 mya [56].

The divergence time estimated between *C. clarum* and *C. castanotum* in this study can be viewed in context of *Cinclosoma* species’ divergences within mesic, semi-arid and arid environments across Australia and New Guinea generally [17]. A species tree estimated in *BEAST [57] using eight nuclear markers (common to this study) and a slightly lower evolutionary rate (0.0207 mutations per million year versus an *ND2* rate of 0.029 mutations per million year in this study) inferred that semi-arid and arid species of *Cinclosoma* diverged from the ancestor of the eastern Australian mesic species *C. punctatum* 17.67 mya. Semi-arid species (*C. clarum* and *C. castanotum*) and the remaining four arid species diverged from each other 13.8 mya. The divergence between the semi-arid species (*C. clarum* and *C. castanotum*) was inferred as 2.1 mya, earlier than the estimate from this study. This difference may be reconciled by i) *BEAST not accounting for gene flow between populations, or ii) limitations of the methods. Gene flow estimates in the current study were low, and, if anything, a species tree discounting gene flow would appear shallower than one with positive levels of gene flow. The answer is most likely to be in the limitations of the methods, e.g., number of populations that can be studied, differences in models of nucleotide evolution. Interestingly, a comparison between a Poisson random field (PRF) method and *BEAST divergence time estimates across the Isthmus of Panama for 22 bird sister pairs reported that *BEAST almost consistently estimated deeper divergence times [58]. The continued improvement of these and related methods will facilitate improved parameter estimates in the future [59].

Next, estimates of rates of migration since divergence also clarified the history of speciation between *C. clarum* and *C. castanotum*. The model based on a coalescent migration rate of zero from *C. clarum* to *C. castanotum* could not be rejected. The effective number of migrants per generation since divergence in the reverse direction was significantly greater than zero, but low (2 Nm = 0.238). This level of migration since divergence (2 Nm <1) is not enough to maintain cohesion of a single species of quail-thrush and is consistent with current species delimitation of *C. clarum* and *C. castanotum*. The similarly low migration rate estimates from analyses repeated without the mtDNA locus are noteworthy in light of evidence from elsewhere of mtDNA divergence despite nuclear gene flow in the Eastern Yellow Robin *Eopsaltria australis* [14, 15]. In that case mtDNA divergence was driven by positive and purifying selection on the mitochondrial genome, but here we confirm results are genome wide and more likely reflect demographic processes.

In using the methodologies herein we have robustly tested the species rank recently accorded to *C. clarum* and *C. castanotum* [9] using multiple-step, species delimitation methodology [19–21]. A population clustering method (GENELAND) using multi-locus allelic data was used to identify distinct populations without prior assignment of individuals to populations. A coalescent-based ‘isolation with migration’ model was used to confirm that migration since divergence has been low enough to prevent breakdown of species barriers and therefore maintain distinct species.

Bayesian inference of population size history (EBSP) suggested that a significant population expansion event in *C. clarum* began before the LGM. *C. clarum* began to expand ca. 50 kya and continued to expand through the Holocene until the present. The signature of population increase in *C. castanotum* was more recent, around 20 kya, and not as substantial. It is only evident in the mean and highest posterior density and not clear according to lowest posterior density. This may be a power-affect due to fewer individuals sampled in this species. More substantial population expansion in *C. clarum* compared with C. *castanotum* according to EBSP is consistent with earlier results from mtDNA summary statistics (Ramos and Onsin’s *R*_*2*_ and Fu’s *Fs*) suggesting statistically significant expansion to the west of the Eyrean Barrier in *C. clarum*, but not to the east of the Eyrean Barrier in *C. castanotum* [9]. Notably, the timing of population expansion in both species are broadly concordant with the timing in butcherbirds [5] where estimates for western grey butcherbirds (between 30 and 25 ka), eastern grey butcherbirds (from 50 ka) and 26 of 32 Australian avian populations which showed signatures of population expansion just prior to the LGM, ca 35 ka (19–68 ka).

Similar geographical patterns of greater expansion west of the Eyrean Barrier than to its east have been observed in ringneck parrots *Barnardius* spp. [47] and Variegated Fairy-wrens *Malurus lamberti* [60] though these patterns were not rigorously dated. This pattern may reflect an interaction between habitat (these are all birds of mallee woodlands) and, simply, the far greater area of that habitat that is available to populations west of the Eyrean Barrier (Fig. 1). The asymmetric pattern of gene flow we observed (greater from *C. castanotum* into *C. clarum* than the reciprocal) may reflect fine scale habitat availability in the region of the southern Flinders Ranges-Eyre Peninsula. It warrants closer attention through finer scale sampling of the populations of *C. clarum fordianum* in that region than was possible in this study. We are pursuing that work from museum specimen-based genomic data.

A key objective of this study was to examine the historical demography that accompanied speciation of these two quail-thrush. Notably, we found that the effective population size of the ancestor of *C. clarum* and *C. castanotum* was substantially reduced compared with current population sizes. The joint posterior estimate of the ancestral effective population size was 2287, which is 54 times (*C. castanotum*) to 178 times (*C. clarum*) lower than joint posterior estimates of current population. Current effective population sizes on a demographic scale (123,031 for *C. castanotum* and 406,598 for *C. clarum*) seem large compared to these species’ current conservation status of vulnerable, near threatened or rare (depending on state/territory) (Atlas of Living Australia accessed 4 January 2016). There are several assumptions used in the analyses which may contribute to this discrepancy: i) effective population sizes are assumed to be constant in each time period (pre- and post-divergence); and ii) potential error in mutation rate estimates used for *ND2* (mtDNA) and/or generation time. Estimates may reflect effective population sizes prior to more recent population declines due to habitat loss, fragmentation and degradation post-European settlement [61–63]. Results from EBSP demographic analyses suggest the violation of the assumption of constant effective population size in each time period (at least in *C. clarum* in the time period since divergence). Notwithstanding these problems of model-fitting, there is a consistent and clear signal of a large reduction in population size in the speciation history of these birds, whether it is at the time of population splitting or post-divergence, followed by significant population expansion.

Available data suggest a dynamic history of vegetation assemblages inhabited by these birds in semi-arid southern Australia, one that is consistent with our findings. Studies in south-eastern Australia suggest a progression from species-rich sclerophyll in the Early Pleistocene, to reduced tree-cover in the Middle Pleistocene, to herb fields and chenopod shrubs dominating the Late Pleistocene to initial Holocene [54, 64, 65]. By the early Holocene, tall trees and shrubs returned to form the current semi-arid adapted woodland as broadly evident today [54]. Following divergence, EBSP analyses suggest that these species had (or continued to have) restricted effective population sizes in the Late Pleistocene, probably due to the lack of tall trees and shrubs and dominance of herbs and chenopod shrubs at that time. Population expansion most likely coincided with the very recent increase in numbers of tall trees and shrubs transitioning into the semi-arid adapted woodland evident today.

A comparable multi-locus study of population demography associated with divergence across southern Australia in birds, albeit of an estuarine and aquatic species, is that between south-west and south-east populations of the Chestnut Teal (*Anas castanea*) [66]. This divergence was more recent at 0.21 mya, was subject to higher numbers of effective migrants per generation since divergence (2 Nm = 16 from southeast into southwest and 2 Nm = 1.0 from southwest into southeast), and notably was not associated with reduction in population size in the history of divergence according to IMa2. In the Chestnut Teal, the southwest population may have contracted since divergence, while the southeast population remained stable or expanded from an effective population size of 400,000. These demographic factors (more recent divergence, higher gene flow and higher ancestral population size) may well explain the basis for the lack of phenotypic divergence in the Chestnut Teal compared with moderate differentiation between *C. clarum* and *C. castanotum.* Further, roles of Z-linked loci and sexual selection in the divergence of the Chestnut Teal from its close relative the Grey Teal *A. gracilis* are now apparent in that case [67]. Clearly, the roles of natural and sexual selection need to be explored in the moderate differentiation between *C. clarum* and *C. castanotum*. More multi-locus data sets from species that have undergone phenotypic divergence, like these quail-thrush, such as the Blue Bonnet and Naretha Bluebonnet (*Northiella haematogaster* and *N. narethae*, respectively) and those that have remained relatively more stable phenotypically, despite substantial time since divergence, such as the White-eared Honeyeater (*Nesoptilotis leucotis*) and the Scarlet Robin *Petroica boodang* [8, 9] are required to more fully partition which processes drove these varying outcomes of Pleistocene divergence across southern Australia. We also draw attention to the different patterns of sexual dimorphism in *C. castanotum* and *C. clarum* [9] and so the worth of exploring a role of sexual selection in their differentiation. This study indicates that drastic reductions in population size associated with isolation and expansion into newly available habitat could have been among the key drivers or, at least, correlates of corresponding phenotypic divergence across southern semi-arid Australia.

## Conclusions

Multi-locus data are important for understanding the processes involved in divergence and for improving understanding of why some species differentiate and maintain integrity as separate species, while others subjected to apparently the same climatic regime continue to be a single cohesive species. Here we posed the question, when speciation does occur, is it accompanied by increased genetic drift due to large reductions in population size when in contracted refugia, colonisation of newly suitable habitat, or divergent selection? While further research is required to unravel the relative roles of natural and sexual selection in this case, differing patterns of sexual dimorphism in the two species suggests a role for sexual selection operating in concert with variation in effective population size. Certainly, a role for drift is consistent with our findings. As it becomes more common to obtain data from a larger number of loci through methods such as exon capture and RAD sequencing approaches (e.g. [68, 69]), as well as whole genome approaches to estimate effective population size over 10 mya [70], the power to obtain demographic parameters associated with divergence will improve. Integration of this with species’ ecology, such as the mallee habitats of the species studied here, and palaeoenvironmental history of different continents and regions will likely also be important in explaining patterns of concordance and discordance among species. For example, numerous bird species in Australia now appear to have undergone expansion prior to the LGM [7]. Yet large post-LGM declines in population size are not apparent in the quail-thrush studied here and the Grey Butcherbird *Cracticus torquatus* [5], all sedentary birds of semi-arid woodlands and shrublands. That such a decline has been reported in the Budgerigar *Melopsittacus undulatus*, a small nomadic parrot of similar habitats in inland Australia, may reflect its very different ecology tied to free-standing water. Alternatively, it may be related to the increased power of the whole genome analysis from which that decline was inferred in that species [70].

## Additional files

Additional file 1: Table S1. Museum registration and location details of specimens used in this study are listed. (PDF 187 kb)
Additional file 2: Figure S1. Identification of genetic discontinuities using GENELAND: analyses of allelic data from 11 autosomal nuclear loci reveal two distinct genetic clusters either side of the Eyrean Barrier. Posterior densities for *k* = 1 to 4 clusters and maps of posterior probability to belong to cluster 1 or 2. (PDF 173 kb)

## Abbreviations

bp: Base pairs
EBSP: Extended Bayesian Skyline Plot
hABC: Hierarchical Approximate Bayesian Computation
HKA: Hudson-Kreitman-Aguade
HKY: Hasegawa, Kishino and Yano
HPD: Highest Posterior Density
indels: Insertions or deletions
kya: Thousand years ago
LGM: Last Glacial Maximum
mtDNA: Mitochondrial DNA
mya: Million years ago
nDNA: Nuclear DNA
RAD: Restriction site Associated DNA markers
