# Supplementary material for: Multi-locus sequence data illuminate demographic drivers of Pleistocene speciation in semi-arid southern Australian birds (Cinclosoma spp.)
Source: BMC Evol Biol. 2016 Oct 22;16:226. doi: 10.1186/s12862-016-0798-6 (PMC5075194; doi:10.1186/s12862-016-0798-6)
Supplement: Additional file 1: Table S1. — Museum registration and location details of specimens used in this study are listed. (PDF 187 kb) [file 12862_2016_798_MOESM1_ESM.pdf]

TissueID	Species	Museum	State	Locality	Latitude	Longitude
22722	Cinclosoma clarum	ANSP	SA	Yardea, Gawler Ranges	-32.417	135.567
22730	Cinclosoma clarum	ANSP	SA	Yardea, Gawler Ranges	-32.417	135.567
22732	Cinclosoma clarum	ANSP	SA	Yardea, Gawler Ranges	-32.417	135.567
51838	Cinclosoma clarum	ANWC	SA	Shed Tank Camp 91 km N of CooK	-29.891	130.093
51855	Cinclosoma clarum	ANWC	SA	18.5 km N of Shed Tank Camp ca 100 km N of Cook	-29.724	130.12
51857	Cinclosoma clarum	ANWC	SA	38.9 km N of Shed Tank camp, c.130 km N of Cook	-29.7253	130.1203
51955	Cinclosoma clarum	ANWC	SA	5.4 km E of Vokes Hill Corner Anne Beadell Highway	-28.577	130.764
51956	Cinclosoma clarum	ANWC	SA	5.4 km E of Vokes Hill Corner Anne Beadell Highway	-28.577	130.764
52039	Cinclosoma clarum	ANWC	SA	12.2 km S of Vokes Hill Corner ca 254 km N of Cook	-28.638	130.603
52040	Cinclosoma clarum	ANWC	SA	12.2 km S of Vokes Hill Corner ca 254 km N of Cook	-28.638	130.603
52041	Cinclosoma clarum	ANWC	SA	18.1 km S of Vokes Hill Corner ca 247 km N of Cook	-28.657	130.557
52080	Cinclosoma clarum	ANWC	SA	Irish Well 21 km SW of Highway between Oak Valley/Maralinga	-29.959	131.093
52088	Cinclosoma clarum	ANWC	SA	6 km NE of Irish Well ca 48 km NW of Maralinga	-29.953	131.074
52089	Cinclosoma clarum	ANWC	SA	6 km NE of Irish Well ca 48 km NW of Maralinga	-29.953	131.074
52266	Cinclosoma clarum	ANWC	SA	Yellabinna Area 86.6 km NNE of Ceduna	-31.554	133.978
54215	Cinclosoma clarum	ANWC	WA	91.8 km E of Ilkurlka Great Victoria Desert	-28.534	128.281
54218	Cinclosoma clarum	ANWC	WA	96.5 km E of Ilkurlka Great Victoria Desert	-28.534	128.329
54225	Cinclosoma clarum	ANWC	WA	70.4 km E of Ilkurlka Great Victoria Desert	-28.456	128.091
54232	Cinclosoma clarum	ANWC	WA	48.6 km E of Ilkurlka Great Victoria Desert	-28.36	127.957
54234	Cinclosoma clarum	ANWC	WA	56.6 km E of Ilkurlka Great Victoria Desert	-28.403	128.018
55864	Cinclosoma clarum	SAMA	SA	Yalata ca 67 km WNW Yalata on Coombra Track	-31.389	131.174
33368	Cinclosoma clarum	ANWC	WA	Tamala Stn S of Shark Bay	-26.627	113.833
28857	Cinclosoma castanotum	ANWC	NSW	Nombinnie Nature Reserve ca 3 km NW of Lake Cargelligo	-33.091	146.215
28858	Cinclosoma castanotum	ANWC	NSW	Round Hill Nature Reserve ca 30 km SE of Mount Hope	-33.039	146.202
42435	Cinclosoma castanotum	ANWC	SA	Goondooloo 31 km W of Halidon 110 km E of Adelaide	-34.867	139.817
48273	Cinclosoma castanotum	ANWC	SA	26 km N of Arkaroola Station N Flinders Range	-30.209	139.23
48274	Cinclosoma castanotum	ANWC	SA	26 km N of Arkaroola Station N Flinders Range	-30.209	139.23
49644	Cinclosoma castanotum	ANWC	NSW	Round Hill Nature Reserve ca 30 km SE of Mount Hope	-32.961	146.111
49654	Cinclosoma castanotum	ANWC	NSW	Round Hill Nature Reserve ca 30 km SE of Mount Hope	-32.961	146.111

ANWC : Australian National Wildlife Collection, CSIRO National Research Collections

ANSP : Academy of Natural Sciences at Drexel University, Philadelphia

SAMA : South Australian Museum, Adelaide
