# Supplementary figures and images for: Multi-locus sequence data illuminate demographic drivers of Pleistocene speciation in semi-arid southern Australian birds (Cinclosoma spp.)
Source: BMC Evol Biol. 2016 Oct 22;16:226. doi: 10.1186/s12862-016-0798-6 (PMC5075194; doi:10.1186/s12862-016-0798-6)

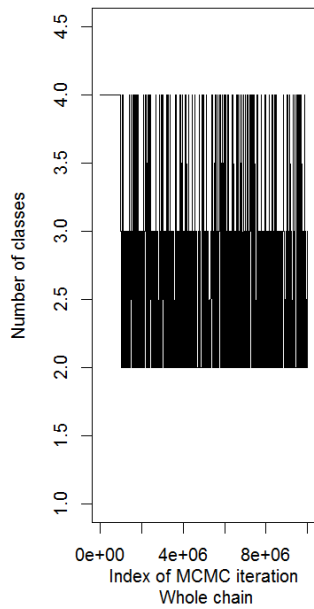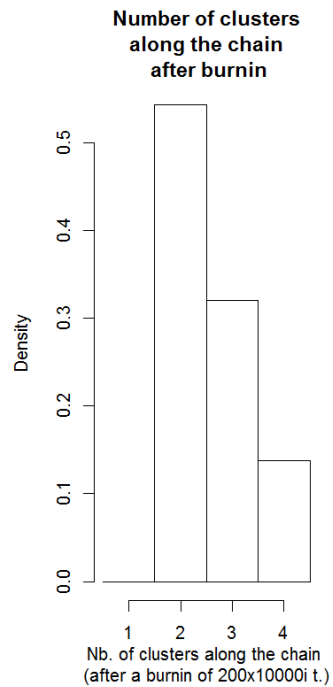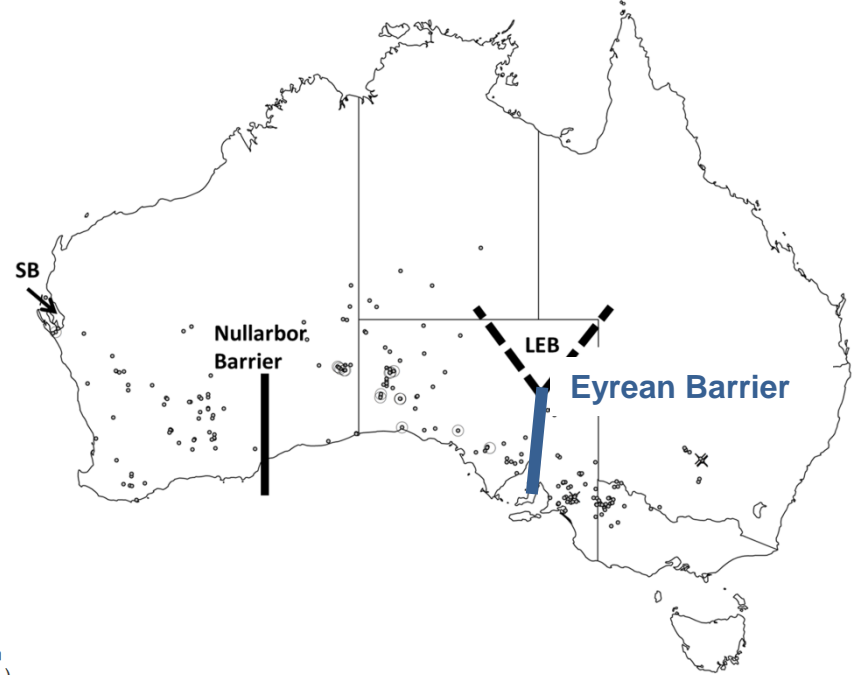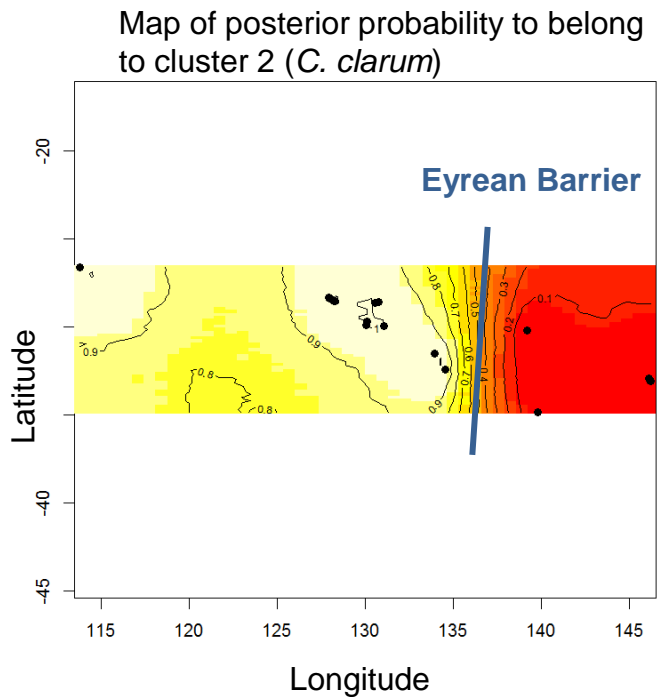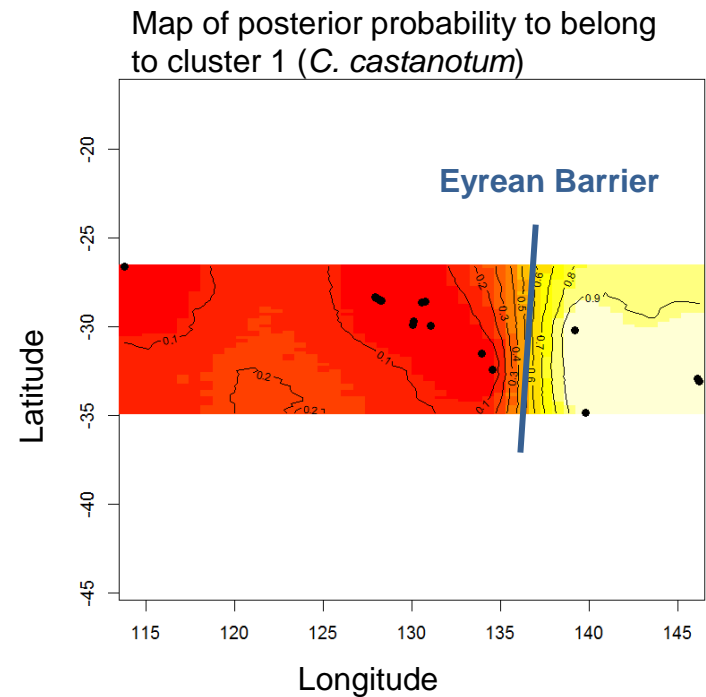

Supplement: Additional file 2: Figure S1. — Identification of genetic discontinuities using GENELAND: analyses of allelic data from 11 autosomal nuclear loci reveal two distinct genetic clusters either side of the Eyrean Barrier. Posterior densities for k = 1 to 4 clusters and maps of posterior probability to belong to cluster 1 or 2. (PDF 173 kb) [file 12862_2016_798_MOESM2_ESM.pdf]
